# Single-cell RNA sequencing reveals spatial heterogeneity and immune evasion of circulating tumor cells

**DOI:** 10.20892/j.issn.2095-3941.2021.0466

**Published:** 2021-11-24

**Authors:** Yunfan Sun, Jian Zhou, Jia Fan, Xinrong Yang

**Affiliations:** 1 Department of Liver Surgery & Transplantation, Liver Cancer Institute, Zhongshan Hospital, Fudan University; Key Laboratory of Carcinogenesis and Cancer Invasion, Ministry of Education, Shanghai 200032, China

Circulating tumor cells (CTCs), recognized as intermediaries between primary and distant lesions, are key to understanding the mechanisms of cancer metastasis. Because the dissemination of tumor cells in the circulatory system is a spatially and temporally dynamic process, CTCs can logically be assumed to be a population of cells displaying both spatial and temporal heterogeneity^1^. Prior studies have revealed substantial temporal heterogeneity in CTC phenotypes during anti-cancer treatments^2,3^. However, current knowledge of CTCs is derived mostly from peripheral venous blood; thus, only a snapshot of the entire circulatory process has been determined, and much more remains to be understood.

In the past few years, we have extensively investigated the spatiotemporal heterogeneity of CTCs in our laboratory. Our exploratory journey started from systemically mapping CTC distribution and characterizing their epithelial-to-mesenchymal transition (EMT) features across multiple vascular compartments in patients with localized hepatocellular carcinoma (HCC). The cellular number and size gradient between tumor efferent vessels and post-pulmonary vessels was remarkable. Tracking the fate of CTC clusters revealed that CTCs spread in a singular-aggregated manner. Characterization of EMT features demonstrated that CTCs are predominantly epithelial at release, but the EMT program is dynamically activated during hematogeneous transit. Moreover, the CTC burden in hepatic veins and peripheral circulation is predictive of postoperative lung metastasis and intrahepatic recurrence, respectively. A pilot study published in *Clinical Cancer Research*^4^ has demonstrated profound spatiotemporal heterogeneity in cellular distribution, EMT features, and clinical significance among CTCs. On this basis, we further hypothesized that, to survive the inhospitable circulatory microenvironment and colonize distant sites, CTCs might spatially and temporally modulate their phenotypic characteristics and molecular signaling by altering the transcriptional program during dissemination.

**Figure 1:**
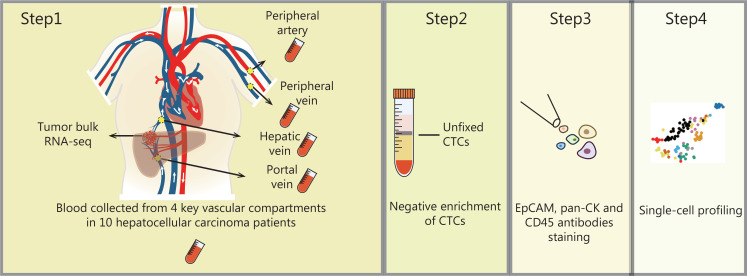
Overview of the workflow for CTC isolation and single-cell RNA preparation.

To address this possibility, in our recent study published in *Nature Communications*, we established single-cell RNA sequencing (scRNA-seq) profiles of individual CTCs isolated from 4 key vascular sites along the HCC hematogenous metastatic pathway—the hepatic vein (HV), peripheral artery (PA), peripheral vein (PV), and portal vein (PoV)—in 10 localized HCCs (**Figure 1**).

Our data demonstrated significant variations in inter-CTC heterogeneity among vascular compartments. A remarkably heterogeneous CTC population was identified in liver efferent vessels (HV), thus supporting the hypothesis that CTCs are shed randomly from several spatially distinct regions of primary tumors. In the PA, the intercellular heterogeneity of CTCs was significantly diminished, thereby implicating a selection process wherein smaller and more deformable cells preferentially pass through the pulmonary capillary filter. Notably, the intercellular heterogeneity of CTCs was found to be elevated in the PV and PoV. Given the complexity of the bloodstream microenvironment, CTCs are exposed to various biophysical stresses during peripheral circulation, including flow-based shear stresses, loss of anchorage, and interactions between cytokines and immune cells^5,6^. To restore cellular homeostasis, CTCs might activate adaptive stress response pathways that not only increase stress tolerance but also may significantly contribute to their phenotypic diversity. Accordingly, our data showed that the biological processes involved in cell cycling, the immune response, the regulation of cytokine production, and responses to stimuli are specifically enriched in PV CTCs. Thus, CTCs in tumor efferent vessels may represent intratumoral heterogeneity, whereas the transcriptional diversity observed in peripheral CTCs may indicate their adaptation-related evolution.

**Figure 2:**
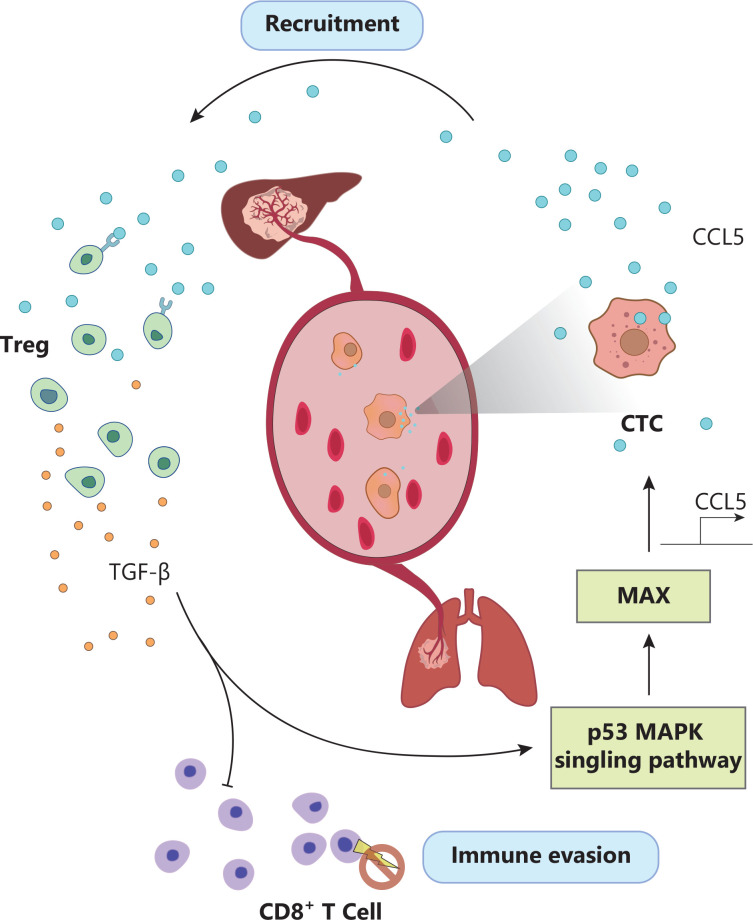
Schematic diagram of how CTCs create an immunosuppressive milieu through hyperactivation of the p38-MAX-CCL5 axis for the recruitment of Treg cells.

After CTCs leave the protective immunosuppressive tumor microenvironment, they are outnumbered by peripheral immune effector cells. The successful evasion of immune-mediated killing is critical for CTC survival and dissemination. In agreement with recent reports^6^, our scRNA-seq data showed that CTCs from patients with HCC exploit a variety of immune-evasion strategies, including EMT, platelet-CTC aggregates, and the production of immunosuppressive chemokines. Excitingly, a novel immune evasion mechanism of CTCs was discovered in this study. We identified CCL5 as a top upregulated gene among 15 immune escape-related genes in CTCs. This gene has previously been implicated in regulatory T cell (Treg) recruitment in other types of cancer. We first confirmed a positive correlation between CCL5^+^ CTCs and peripheral Tregs in 2 independent cohorts of patients with HCC: patients with a high percentage of Tregs and elevated CCL5^+^ CTCs counts in their peripheral blood had a higher risk of recurrence and poorer overall survival. Both *in vitro* and *in vivo* models rigorously validated that CTC-derived CCL5 recruits Tregs, thereby facilitating an immuno-suppressive and pro-metastatic microenvironment in HCC. Further mechanistic studies revealed that the overexpression of CCL5 is transcriptionally regulated by p38-MAX signaling. Knockdown of MAX was found to significantly inhibit tumor growth and distant metastasis of HCC cells, and Treg recruitment in metastatic lesions was abrogated by MAX silencing. Finally, we discovered that peripheral Treg-derived TGF-β1 in turn activates p38-MAX signaling, thus inducing CCL5 expression in CTCs. This reciprocal activation of CTC and Tregs promotes CTC evasion of immune surveillance, thereby enhancing their survival in the bloodstream and facilitating distant metastasis (**Figure 2**).

In summary, our scRNA-seq CTC data reveal remarkable intra- and inter-vascular heterogeneity among CTCs from 4 vascular sites. By comparing CTCs from neighboring vascular sites, we identified the transcriptional dynamics associated with the stress response, cell cycle and immune evasion signaling during transport through consecutive vascular compartments. Our study proposes the use of scRNA-seq to study the mechanism of metastasis on the basis of CTC transcriptomic profiles, which may aid in designing new anti-metastasis therapeutic strategies for HCC.
